# 2,7-Dimethyl-1,8-naphthyridine

**DOI:** 10.1107/S1600536809024350

**Published:** 2009-07-04

**Authors:** Hoong-Kun Fun, Chin Sing Yeap, Nirmal Kumar Das, Ajit Kumar Mahapatra, Shyamaprosad Goswami

**Affiliations:** a X-ray Crystallography Unit, School of Physics, Universiti Sains Malaysia, 11800 USM, Penang, Malaysia; b Department of Chemistry, Bengal Engineering and Science University, Shibpur, Howrah 711 103, India

## Abstract

The asymmetric unit of the title compound, C_10_H_10_N_2_, contains one half-mol­ecule with the two shared C atoms lying on a twofold rotation axis. The 1,8-naphthyridine is almost planar with a dihedral angle of 0.42 (3)° between the fused pyridine rings. In the crystal, mol­ecules are linked into infinite chains along the *c* axis by inter­molecular C—H⋯N hydrogen bonds, generating *R*_2_^2^(8) ring motifs. In addition, the crystal structure is further stabilized by C—H⋯π inter­actions.

## Related literature

For applications of naphthyridines, see: Badawneh *et al.* (2001 ▶); Hawes *et al.* (1977 ▶); Gorecki & Hawes (1977 ▶). For mol­ecular recognition chemistry of naphthyridines, see: Goswami & Mukherjee (1997 ▶); Goswami *et al.* (2001 ▶, 2005 ▶). For the preparation of 2,7-dimethyl-[1,8]naphthyridine, see: Chandler *et al.* (1982 ▶). For hydrogen-bond motifs, see: Bernstein *et al.* (1995 ▶). For the stability of the temperature controller used in the data collection, see: Cosier & Glazer (1986 ▶).
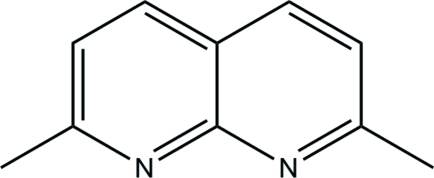


## Experimental

### 

#### Crystal data

C_10_H_10_N_2_
*M* *_r_* = 158.20
Orthorhombic, 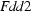

*a* = 13.3977 (2) Å
*b* = 19.3492 (4) Å
*c* = 6.3089 (1) Å
*V* = 1635.49 (5) Å^3^
*Z* = 8
Mo *K*α radiation
μ = 0.08 mm^−1^
*T* = 100 K
0.57 × 0.41 × 0.24 mm

#### Data collection

Bruker SMART APEXII CCD area-detector diffractometer
Absorption correction: multi-scan (**SADABS**; Bruker, 2005 ▶) *T*_min_ = 0.939, *T*_max_ = 0.981
15454 measured reflections
1153 independent reflections
1116 reflections with *I* > 2σ(*I*)
*R*_int_ = 0.024

#### Refinement

*R*[*F* ^2^ > 2σ(*F* ^2^)] = 0.034
*wR*(*F* ^2^) = 0.098
*S* = 1.09
1153 reflections
57 parameters
1 restraint
H-atom parameters constrained
Δρ_max_ = 0.51 e Å^−3^
Δρ_min_ = −0.25 e Å^−3^

### 

Data collection: *APEX2* (Bruker, 2005 ▶); cell refinement: *SAINT* (Bruker, 2005 ▶); data reduction: *SAINT*; program(s) used to solve structure: *SHELXTL* (Sheldrick, 2008 ▶); program(s) used to refine structure: *SHELXTL*; molecular graphics: *SHELXTL*; software used to prepare material for publication: *SHELXTL* and *PLATON* (Spek, 2009 ▶).

## Supplementary Material

Crystal structure: contains datablocks global, I. DOI: 10.1107/S1600536809024350/bq2147sup1.cif

Structure factors: contains datablocks I. DOI: 10.1107/S1600536809024350/bq2147Isup2.hkl

Additional supplementary materials:  crystallographic information; 3D view; checkCIF report

## Figures and Tables

**Table 1:** Hydrogen-bond geometry (Å, °)

*D*—H⋯*A*	*D*—H	H⋯*A*	*D*⋯*A*	*D*—H⋯*A*
C3—H3*A*⋯N1^i^	0.93	2.56	3.4889 (9)	175
C6—H6*C*⋯*Cg*1^ii^	0.96	2.78	3.5742 (8)	140
C6—H6*C*⋯*Cg*2^iii^	0.96	2.78	3.5742 (8)	140

Symmetry codes: (i) 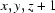; (ii) 
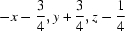
; (iii) 
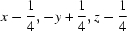
. *Cg*1 and *Cg*2 are the centroids of the N1/C1–C5 and C1–C2/C3*A*–C5*A*/N1*A* rings, respectively.

**Fig. 1.**
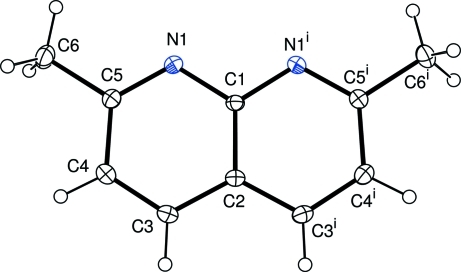
The molecular structure of the title compound with atom labels and 50% probability ellipsoids for non-H atoms. Symmetry code: (i) -x, -y, z.

**Fig. 2.**
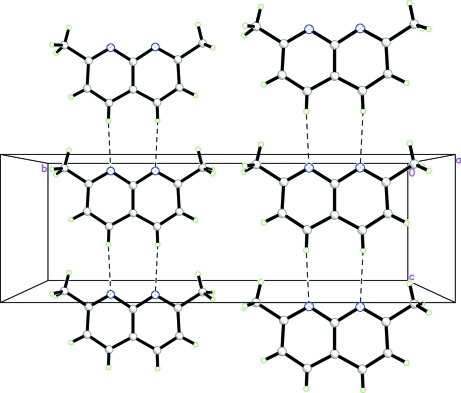
The crystal packing of (I), viewed down the a axis, showing the molecules are linked along the c axis. Intermolecular hydrogen bonds are shown in as dashed lines.

## supplementary crystallographic information

### Comment

Due to their wide applications in medicine, naphthyridines are one of the most useful group of compounds. They are used as antihypertensives, antitumor agents, immunostimulants and herbicide safeners (Badawneh *et al.*, 2001; Hawes *et al.*, 1977; Gorecki *et al.*, 1977). Naphthyridines are also used as a key molecule in molecular recognition chemistry (Goswami & Mukherjee, 1997; Goswami *et al.*, 2005; 2001; Sheldrick, 2008). We report here the single crystal X-ray structure.

In the title compound (I), (Fig. 1), the C1 and C2 atoms are lying on twofold rotation axis [symmetry code: -*x*, -*y*, *z*]. The dihedral angle between the two pyridine rings is equal to 0.42 (3)° indicating that the 1,8-naphthyridine is almost planar. The molecules are linked together into infinite chains by the intermolecular C3—H3A···N1 hydrogen bonds along the *c* axis (Fig. 2) generating *R*_2_^2^(8) ring motifs (Bernstein *et al.*, 1995). The crystal structure is further stabilized by the C—H···π interactions (Table 1).

### Experimental

2,7-dimethyl-[1,8]naphthyridine was prepared according to the literature procedure (Chandler *et al.*, 1982). In a sample bottle, 10 mg of compound was taken and dissolved in CHCl_3_ and by slow evaporation the crystals are formed as colorless blocks.

### Refinement

All hydrogen atoms were positioned geometrically with a riding model approximation with C—H = 0.93-0.96 Å and U_iso_(H) = 1.2 U_eq_(C). The rotating-group model was applied for the methyl groups. As there are not enough anomalous dispersion to determine the absolute structure, 923 Friedel pairs were merged before the final refinement.

### Crystal data

**Table:**

C_10_H_10_N_2_	*F*(000) = 672
*M**_r_* = 158.20	*D*_x_ = 1.285 Mg m^−^^3^
Orthorhombic, *F**d**d*2	Mo *K*α radiation, λ = 0.71073 Å
Hall symbol: F 2 -2d	Cell parameters from 9922 reflections
*a* = 13.3977 (2) Å	θ = 3.0–40.6°
*b* = 19.3492 (4) Å	µ = 0.08 mm^−^^1^
*c* = 6.3089 (1) Å	*T* = 100 K
*V* = 1635.49 (5) Å^3^	Block, colourless
*Z* = 8	0.57 × 0.41 × 0.24 mm

### Data collection

**Table:**

Bruker SMART APEXII CCD area-detector diffractometer	1153 independent reflections
Radiation source: fine-focus sealed tube	1116 reflections with *I* > 2σ(*I*)
graphite	*R*_int_ = 0.024
φ and ω scans	θ_max_ = 37.5°, θ_min_ = 3.7°
Absorption correction: multi-scan (*SADABS*; Bruker, 2005)	*h* = −21→22
*T*_min_ = 0.939, *T*_max_ = 0.981	*k* = −32→31
15454 measured reflections	*l* = −10→10

### Refinement

**Table:**

Refinement on *F*^2^	Primary atom site location: structure-invariant direct methods
Least-squares matrix: full	Secondary atom site location: difference Fourier map
*R*[*F*^2^ > 2σ(*F*^2^)] = 0.034	Hydrogen site location: inferred from neighbouring sites
*wR*(*F*^2^) = 0.098	H-atom parameters constrained
*S* = 1.09	*w* = 1/[σ^2^(*F*_o_^2^) + (0.0689*P*)^2^ + 0.3523*P*] where *P* = (*F*_o_^2^ + 2*F*_c_^2^)/3
1153 reflections	(Δ/σ)_max_ < 0.001
57 parameters	Δρ_max_ = 0.51 e Å^−^^3^
1 restraint	Δρ_min_ = −0.25 e Å^−^^3^

### Special details

**Table:**

Experimental. The crystal was placed in the cold stream of an Oxford Cyrosystems Cobra open-flow nitrogen cryostat (Cosier & Glazer, 1986) operating at 100.0 (1)K.
Geometry. All esds (except the esd in the dihedral angle between two l.s. planes) are estimated using the full covariance matrix. The cell esds are taken into account individually in the estimation of esds in distances, angles and torsion angles; correlations between esds in cell parameters are only used when they are defined by crystal symmetry. An approximate (isotropic) treatment of cell esds is used for estimating esds involving l.s. planes.
Refinement. Refinement of F^2^ against ALL reflections. The weighted R-factor wR and goodness of fit S are based on F^2^, conventional R-factors R are based on F, with F set to zero for negative F^2^. The threshold expression of F^2^ > 2sigma(F^2^) is used only for calculating R-factors(gt) etc. and is not relevant to the choice of reflections for refinement. R-factors based on F^2^ are statistically about twice as large as those based on F, and R- factors based on ALL data will be even larger.

### Fractional atomic coordinates and isotropic or equivalent isotropic displacement parameters (Å^2^)

**Table:**

	*x*	*y*	*z*	*U*_iso_*/*U*_eq_	
N1	0.01020 (4)	0.05928 (3)	0.32666 (9)	0.01333 (14)	
C1	0.0000	0.0000	0.44228 (13)	0.01141 (18)	
C2	0.0000	0.0000	0.66711 (15)	0.01275 (18)	
C3	0.01175 (6)	0.06389 (4)	0.77384 (11)	0.01521 (15)	
H3A	0.0129	0.0658	0.9211	0.018*	
C4	0.02138 (6)	0.12274 (4)	0.65545 (14)	0.01597 (16)	
H4A	0.0289	0.1653	0.7218	0.019*	
C5	0.01984 (5)	0.11846 (4)	0.42997 (11)	0.01352 (15)	
C6	0.02718 (6)	0.18360 (4)	0.30155 (15)	0.01878 (15)	
H6A	0.0381	0.1721	0.1554	0.028*	
H6B	0.0819	0.2110	0.3525	0.028*	
H6C	−0.0338	0.2094	0.3147	0.028*	

### Atomic displacement parameters (Å^2^)

**Table:**

	*U*^11^	*U*^22^	*U*^33^	*U*^12^	*U*^13^	*U*^23^
N1	0.0165 (3)	0.0124 (3)	0.0111 (3)	−0.00003 (19)	−0.00022 (18)	0.00128 (16)
C1	0.0132 (4)	0.0123 (4)	0.0087 (4)	0.0005 (3)	0.000	0.000
C2	0.0148 (4)	0.0138 (4)	0.0096 (4)	−0.0002 (3)	0.000	0.000
C3	0.0189 (3)	0.0159 (3)	0.0108 (3)	−0.0010 (2)	0.00012 (19)	−0.0019 (2)
C4	0.0196 (4)	0.0138 (3)	0.0144 (3)	−0.0006 (2)	0.0004 (2)	−0.0025 (2)
C5	0.0150 (3)	0.0124 (3)	0.0131 (3)	0.0001 (2)	−0.0002 (2)	0.0010 (2)
C6	0.0229 (3)	0.0137 (3)	0.0197 (3)	−0.0013 (2)	−0.0009 (3)	0.0038 (2)

### Geometric parameters (Å, °)

**Table:**

N1—C5	1.3238 (8)	C3—H3A	0.9300
N1—C1	1.3662 (7)	C4—C5	1.4251 (11)
C1—N1^i^	1.3662 (7)	C4—H4A	0.9300
C1—C2	1.4184 (13)	C5—C6	1.5017 (10)
C2—C3^i^	1.4165 (9)	C6—H6A	0.9600
C2—C3	1.4165 (9)	C6—H6B	0.9600
C3—C4	1.3678 (10)	C6—H6C	0.9600
			
C5—N1—C1	118.23 (6)	C3—C4—H4A	120.2
N1^i^—C1—N1	115.46 (7)	C5—C4—H4A	120.2
N1^i^—C1—C2	122.27 (4)	N1—C5—C4	122.90 (7)
N1—C1—C2	122.27 (4)	N1—C5—C6	117.83 (6)
C3^i^—C2—C3	123.23 (9)	C4—C5—C6	119.26 (7)
C3^i^—C2—C1	118.39 (4)	C5—C6—H6A	109.5
C3—C2—C1	118.38 (4)	C5—C6—H6B	109.5
C4—C3—C2	118.51 (7)	H6A—C6—H6B	109.5
C4—C3—H3A	120.7	C5—C6—H6C	109.5
C2—C3—H3A	120.7	H6A—C6—H6C	109.5
C3—C4—C5	119.69 (7)	H6B—C6—H6C	109.5
			
C5—N1—C1—N1^i^	179.65 (7)	C1—C2—C3—C4	0.76 (8)
C5—N1—C1—C2	−0.35 (7)	C2—C3—C4—C5	−0.28 (11)
N1^i^—C1—C2—C3^i^	−0.46 (5)	C1—N1—C5—C4	0.88 (11)
N1—C1—C2—C3^i^	179.54 (5)	C1—N1—C5—C6	−177.77 (5)
N1^i^—C1—C2—C3	179.54 (5)	C3—C4—C5—N1	−0.57 (13)
N1—C1—C2—C3	−0.46 (5)	C3—C4—C5—C6	178.06 (6)
C3^i^—C2—C3—C4	−179.25 (8)		

Symmetry codes: (i) −*x*, −*y*, *z*.

### Hydrogen-bond geometry (Å, °)

**Table:**

*D*—H···*A*	*D*—H	H···*A*	*D*···*A*	*D*—H···*A*
C3—H3A···N1^ii^	0.93	2.56	3.4889 (9)	175
C6—H6C···Cg1^iii^	0.96	2.78	3.5742 (8)	140
C6—H6C···Cg2^iv^	0.96	2.78	3.5742 (8)	140

Symmetry codes: (ii) *x*, *y*, *z*+1; (iii) −*x*−3/4, *y*+3/4, *z*−1/4; (iv) *x*−1/4, −*y*+1/4, *z*−1/4.
